# Joint trajectories of obesity and depressive symptoms and risk of incident glaucoma: a 16-year prospective cohort study from the Health and Retirement Study

**DOI:** 10.3389/fnagi.2026.1910500

**Published:** 2026-08-03

**Authors:** XiaoDan Xu, Yuting Hu, Fuju Shi, Jingtao Wu, Yongfeng Wu, Yanfeng Zhu, Jing Yang

**Affiliations:** 1 Department of Ophthalmology, Jianyang Chinese Medicine Hospital, Jianyang, China; 2 Clinical Laboratory, The First Affiliated Hospital of Chengdu Medical College, Chengdu, China; 3 Department of Ophthalmology, North China University of Science and Technology Affiliated Hospital, Tangshan, China; 4 School of Public Health, Chengdu Medical College, Chengdu, China; 5 Experimental Teaching Center of Biotechnology, School of Bioscience and Technology, Chengdu Medical College, Chengdu, China; 6 Key Laboratory of Target Discovery and Protein Drug Development in Major Diseases at Chengdu Medical College of Sichuan Province, Chengdu, China; 7 Irradiation Preservation and Effect Key Laboratory of Sichuan Province, Chengdu, China

**Keywords:** body mass index, cohort study, depression, glaucoma, group-based trajectory modeling

## Abstract

**Background:**

Obesity and depression frequently co-occur in older adults and share pathways potentially relevant to glaucoma, yet their dynamic co-evolution and joint contribution to glaucoma risk remain poorly characterized.

**Objectives:**

This study aimed to identify joint longitudinal trajectories of body mass index (BMI) and depressive symptoms and evaluate their association with incident glaucoma.

**Methods:**

Among 6,154 participants from the Health and Retirement Study (2000–2016) in the United States, we applied group-based multi-trajectory modeling to jointly classify BMI and CESD-8 trajectories over nine waves. Incident glaucoma was self-reported. Cox models estimated hazard ratios (HRs) with 95% confidence intervals (CIs).

**Results:**

Over a median follow-up of 12.0 years (interquartile range 8.0–16.0), 234 participants (3.8%) developed incident glaucoma. Four distinct joint trajectories were identified: Normal weight–Low Depression (23.5%, reference), Overweight–Low Depression (25.3%), Normal weight–High Depression (31.9%), and Obese–Moderate Depression (19.3%). Crude incidence rates per 1,000 person-years ranged from 1.92 (95% CI 1.31–2.71) in the reference group to 4.82 (95% CI 3.75–6.11) in the obese–moderate depression group (log-rank *p* < 0.001). In the fully adjusted model, compared with the reference trajectory, participants in the Overweight–Low Depression (HR 1.61, 95% CI 1.04–2.49), Normal weight–High Depression (HR 1.82, 95% CI 1.19–2.78), and obese–moderate depression (HR 2.52, 95% CI 1.61–3.93) groups had significantly increased glaucoma risk (*p* for trend < 0.001). These associations remained consistent across subgroups and sensitivity analyses.

**Conclusion:**

Adverse joint trajectories of elevated BMI and depressive symptoms are independently and dose-dependently associated with higher glaucoma risk, supporting integrated monitoring of metabolic and psychological health in glaucoma prevention.

## Introduction

Glaucoma is the leading cause of irreversible blindness worldwide. In 2021, approximately 95 million people globally were affected by glaucoma, and this number is projected to increase to 112 million by 2040 as the population ages (Peng et al., 2026). This progressive optic neuropathy characterized by retinal ganglion cell loss and visual field deterioration represents a substantial and growing public health burden, particularly among middle-aged and older adults (Rezkallah et al., 2022; Li et al., 2025). While intraocular pressure (IOP) remains the primary modifiable risk factor and the cornerstone of therapeutic intervention, a significant proportion of patients experience disease progression despite attaining target IOP levels through pharmacological, laser, or surgical modalities (Liu et al., 2025). This clinical phenomenon underscores the critical need to identify IOP-independent mechanisms contributing to retinal ganglion cell degeneration, as current treatment strategies may be incomplete without addressing additional pathophysiological pathways.

Accumulating evidence suggests that metabolic disturbances and psychological factors may contribute to glaucoma pathogenesis through IOP-independent mechanisms (Zhang et al., 2019; Russo et al., 2022). Recent large-scale prospective cohort studies, including analyses from the UK Biobank involving over 100,000 participants, have demonstrated significant associations between obesity, metabolic syndrome, and incident primary open-angle glaucoma (Macri et al., 2025; Paul et al., 2026). Similarly, observational studies across diverse populations have reported elevated prevalence of depression and anxiety among glaucoma patients compared with healthy controls, with Mendelian randomization analyses providing convergent evidence for shared genetic architecture between these neurological and ophthalmological conditions (Yin et al., 2024; Deng and Qin, 2024). The clinical significance of these observations is further emphasized by the well-established bidirectional relationship between obesity and depression, wherein each condition reciprocally increases the risk of the other through shared neurobiological mechanisms (Milaneschi et al., 2019; Prodaniuc et al., 2025; Colozza et al., 2025; Qiu et al., 2025). Common pathophysiological pathways implicated in both conditions include chronic low-grade inflammation with elevated circulating pro-inflammatory cytokines, hypothalamic–pituitary–adrenal axis dysregulation resulting in cortisol hypersecretion, autonomic nervous system dysfunction, and alterations in hypothalamic signaling—all of which may plausibly contribute to retinal and optic nerve damage (Milaneschi et al., 2019; Prodaniuc et al., 2025; Colozza et al., 2025; Qiu et al., 2025; Zhang H. et al., 2024). Recent cross-sectional studies among U.S. adults have further elucidated the interplay between obesity, chronic health conditions, and depression, highlighting the mediating role of healthcare access and the moderating effects of physical activity (Luis, 2026a,b). Theoretical frameworks have emphasized the need to integrate biopsychosocial perspectives when examining the co-occurrence of metabolic and psychological risk factors, particularly in aging populations (Luis et al., 2025). Despite recognition of these isolated risk factors, no population-based longitudinal study has characterized their joint longitudinal trajectories or systematically evaluated their combined effect on glaucoma incidence.

To address this limitation, we employed group-based multi-trajectory modeling to identify distinct longitudinal patterns of body mass index and depressive symptoms over 16 years of follow-up using data from the Health and Retirement Study, a nationally representative cohort of American adults aged 50 years and older (Nagin et al., 2018; Lintuaho et al., 2022; Schrempft et al., 2025; Sonnega et al., 2014). Leveraging this concurrent evolution, we aimed to characterize high-risk phenotypic subgroups—persistent obesity, persistent depression, or their co-occurrence—and quantify the differential associations of these trajectory patterns with incident glaucoma, adjusting for established confounders. Understanding whether persistent co-occurrence of obesity and depression confers additive or synergistic effects on glaucoma development may inform precision risk stratification and guide the design of targeted preventive interventions. Such knowledge has potential implications for reducing the global burden of glaucoma-related blindness through holistic clinical approaches that address modifiable lifestyle factors and psychological well-being in addition to traditional IOP management.

## Materials and methods

### Study design and population

This study used data from the Health and Retirement Study (HRS), an ongoing nationally representative longitudinal cohort of U.S. adults aged 50 years and older, administered by the University of Michigan with biennial interviews collecting information on demographics, health behaviors, chronic conditions, psychosocial status, and functional outcomes. We used nine consecutive waves from Wave 5 (2000) through Wave 13 (2016), with Wave 5 as the baseline and subsequent waves to characterize exposure trajectories and ascertain incident glaucoma. Of 19,578 HRS participants assessed at Wave 5, we sequentially excluded 210 individuals aged below 45 years, 1,102 with missing Center for Epidemiologic Studies Depression Scale (CESD) data, 2,154 with missing body mass index (BMI) data, and 588 with prevalent glaucoma at baseline and 9,370 with unknown glaucoma status during follow-up, yielding a final analytic sample of 6,154 participants who were followed for a maximum of 16 years.

### Assessment of longitudinal exposures

Body mass index was calculated from self-reported body weight in kilograms divided by the square of height in meters (kg/m^2^) at each biennial wave. Depressive symptoms were quantified using the 8-item Center for Epidemiologic Studies Depression Scale (CESD-8), a validated short form widely used in aging cohorts, with total scores ranging from 0 to 8 and a score of 3 or greater commonly considered indicative of clinically elevated depressive symptomatology. Both BMI and CESD-8 were measured repeatedly across Waves 5 through 13, providing up to nine longitudinal observations per participant spanning a 16-year window for joint trajectory modeling. Baseline characteristics of included and excluded participants were compared to assess potential selection bias (Supplementary Table 1).

### Ascertainment of incident glaucoma

Incident glaucoma was ascertained through self-reported physician diagnosis obtained at each follow-up wave. Although self-reported glaucoma diagnosis has been used in prior population-based studies utilizing HRS and similar aging cohorts, this approach may be subject to outcome misclassification and detection bias, as diagnosis depends on access to eye care and frequency of ophthalmic examinations; these limitations are addressed further in the Discussion. Participants with a positive glaucoma history at Wave 5 were excluded, and the first wave at which a participant reported a new physician diagnosis was used to define incident events. Follow-up time was calculated in years from the baseline interview to the date of glaucoma diagnosis, loss to follow-up, death, or the end of observation at Wave 13, whichever occurred first.

### Covariates

Baseline covariates were selected *a priori* based on established associations with obesity, depression, or glaucoma risk, and comprised sociodemographic characteristics (age as a continuous variable, sex, race/ethnicity classified as White, Black, or Other, educational attainment categorized as low [less than high school], medium [high school graduate or equivalent], or high [some college or above], marital status, and residence classified as urban or rural according to the Beale Rural–Urban Continuum Code), lifestyle behaviors (current smoking and current drinking, each coded as yes or no), history of chronic conditions (hypertension, diabetes mellitus, heart disease, stroke, arthritis, chronic lung disease, and cancer, each coded as yes or no based on self-reported physician diagnosis), and baseline self-reported distance and near vision scores [each rated on a scale from 1 (poor) to 4 (excellent)]. Distance and near vision were included as covariates to control for baseline visual function that may independently influence healthcare-seeking behavior and glaucoma detection; the potential for overadjustment was examined in a sensitivity analysis omitting these variables. A complete list of variable definitions and coding schemes is provided in Supplementary Table 2.

### Joint trajectory identification

To jointly characterize the longitudinal co-evolution of BMI and depressive symptoms, we applied group-based multi-trajectory modeling (GBMTM) using the finite mixture of mixed-effects regression framework implemented in the flexmix R package (Leisch, 2004). Specifically, the joint model simultaneously estimated class-specific trajectories for two response variables—BMI and CESD-8—within a single latent class structure, such that each latent class defined a unique pair of BMI and depressive symptom trajectories. Prior to modeling, BMI and CESD-8 were each standardized to z-scores using the pooled mean and standard deviation across all person-wave observations to ensure comparable scale contributions. Time was parameterized as years since baseline (0, 2, 4, …, 16 years), and each trajectory dimension was modeled as a Gaussian response with a quadratic polynomial of time, permitting nonlinear evolution; a linear specification was retained as a fallback if the quadratic model failed to converge. A Gaussian distribution was adopted for the CESD-8 *z*-scores because standardization rendered the transformed scores approximately continuous and unbounded, and simulation studies have shown that Gaussian-based trajectory models yield robust group recovery even when the underlying variable is moderately skewed[ref]. Participants with intermittent missing BMI or CESD-8 values were retained in the model, as the flexmix framework accommodates unbalanced longitudinal data through maximum likelihood estimation under a missing-at-random assumption. The optimal number of trajectory groups was determined by jointly considering the Akaike information criterion (AIC), Bayesian information criterion (BIC), average silhouette width, and gap statistic evaluated over *K* = 2–6, together with clinical interpretability, average posterior probability of assignment (threshold ≥ 0.70), and a minimum group size of 5%. Each participant was assigned to the trajectory group with the highest posterior probability. To quantify classification uncertainty, we reported the mean posterior probability for each class; all classes exceeded the recommended threshold, with values ranging from 0.82 to 0.94 (overall average 0.89; Supplementary Table 3).

### Statistical analysis

Baseline characteristics were summarized as mean (standard deviation) or median (interquartile range) for continuous variables and as counts with percentages for categorical variables. Between-group comparisons were conducted using the Wilcoxon rank-sum test or Student’s *t*-test for continuous variables and the chi-squared test for categorical variables, with standardized mean differences additionally reported to quantify between-group imbalance. Cumulative incidence of glaucoma by trajectory group was estimated using the Kaplan–Meier method and compared with the log-rank test, and crude incidence rates per 1,000 person-years were calculated with 95% confidence intervals derived from the exact Poisson distribution. The association between joint BMI–depression trajectories and incident glaucoma was evaluated using Cox proportional hazards regression, with the healthiest trajectory group (normal weight with low depressive symptoms) specified as the reference. Three hierarchically adjusted models were fitted: Model 1 adjusted for age and sex; Model 2 additionally adjusted for education, marital status, residence, smoking, and drinking; and Model 3 further adjusted for hypertension, diabetes mellitus, heart disease, stroke, arthritis, chronic lung disease, cancer, distance vision, near vision, and race/ethnicity. The proportional hazards assumption was verified using Schoenfeld residuals, and a linear trend across ordered trajectory groups was tested by modeling group membership as an ordinal variable. Prespecified subgroup analyses were conducted according to age (<75 vs. ≥75 years), sex, race/ethnicity (White vs. Black), education, hypertension, diabetes, and smoking status, with multiplicative interactions assessed by likelihood ratio tests comparing nested Cox models with and without product terms. The Other race/ethnicity category was not analyzed separately owing to insufficient sample size and event counts. To evaluate the robustness of findings, sensitivity analyses were performed by (i) excluding incident glaucoma events occurring within the first 2 years of follow-up to address potential reverse causation, (ii) excluding participants with baseline vision impairment, (iii) applying a Fine–Gray subdistribution hazard model treating all-cause mortality as a competing risk (Fine and Gray, 1999), (iv) re-estimating trajectories using a restricted modeling window (Waves 5–9), (v) restricting analyses to complete cases, (vi) excluding individuals with major baseline chronic diseases, (vii) applying a landmark analysis by excluding the first 4 years of follow-up to further mitigate reverse causation bias, (viii) fitting models without adjustment for vision variables to examine whether results were sensitive to potential overadjustment given that visual impairment may lie on the causal pathway between exposures and glaucoma detection, and (ix) additionally adjusting for race/ethnicity to assess potential confounding by racial and ethnic differences in glaucoma susceptibility. All analyses were performed using R version 4.3.0, with two-sided *p* values <0.05 considered statistically significant. The normality of continuous variables was evaluated using the Shapiro–Wilk test, and variables with *p* < 0.05 were summarized as median (interquartile range) with non-parametric tests applied accordingly. Although HRS employs a complex multistage probability sampling design, survey weights were not applied in the present analysis because the trajectory modeling framework in flexmix does not accommodate sampling weights; accordingly, the results should be interpreted as associations within the analytic sample rather than nationally representative estimates. This study was conducted in accordance with the principles of the Declaration of Helsinki and followed the Strengthening the Reporting of Observational Studies in Epidemiology (STROBE) guidelines.

## Results

### Study population

**Figure 1:**
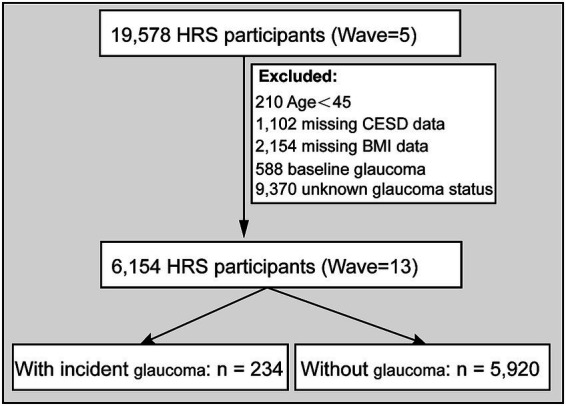
Flow diagram of participant selection from the Health and Retirement Study (HRS). Of 19,578 participants assessed at Wave 5 (baseline, 2000), individuals were sequentially excluded for age below 45 years (*n* = 210), missing Center for Epidemiologic Studies Depression Scale data (*n* = 1,102), missing body mass index data (*n* = 2,154), prevalent glaucoma at baseline (*n* = 588) and unknown glaucoma status during follow-up (*n* = 9,370), yielding a final analytic sample of 6,154 participants, among whom 234 developed incident glaucoma and 5,920 remained glaucoma-free over the follow-up period through Wave 13 (2016).

Of the 19,578 Health and Retirement Study participants assessed at Wave 5 (2000), 6,154 were included in the final analytic sample after sequential exclusions for age below 45 years (*n* = 210), missing CESD data (*n* = 1,102), missing BMI data (*n* = 2,154), and prevalent glaucoma at baseline (*n* = 588) and unknown glaucoma status during follow-up (*n* = 9,370) (Figure 1). Among the 6,154 participants included in the analysis, the median age was 72.0 years [interquartile range (IQR), 68.0–77.0], 59.4% (*n* = 3,657) were women, and the median baseline BMI was 26.10 kg/m^2^ (IQR, 23.50–29.20) (Table 1). The median CESD-8 score was 1.0 (IQR, 0.0–2.0), with 20.6% (*n* = 1,270) meeting the threshold for clinically elevated depressive symptoms (CESD-8 ≥ 3). Compared with excluded participants, the analytic sample had a slightly lower prevalence of stroke (6.6% vs. 7.4%) and chronic lung disease (6.3% vs. 7.1%), though most baseline characteristics were broadly similar (Supplementary Table 1).

**Table 1:** Baseline characteristics of study participants overall and stratified by incident glaucoma status during follow-up.

Characteristics	Level	Overall (*n* = 6,154)	No incident glaucoma (*n* = 5,920)	Incident glaucoma (*n* = 234)	*p*	SMD
Age, years		72.00 [68.00, 77.00]	72.00 [68.00, 77.00]	72.00 [68.00, 77.00]	0.748	0.018
Sex	Female	3,657 (59.4)	3,522 (59.5)	135 (57.7)	0.630	0.037
	Male	2,497 (40.6)	2,398 (40.5)	99 (42.3)		
Race/ethnicity	White	5,356 (87.0)	5,154 (87.1)	202 (86.3)	0.790	0.046
	Black	641 (10.4)	614 (10.4)	27 (11.5)		
	Other	157 (2.6)	152 (2.6)	5 (2.1)		
Education level	Low	1,565 (25.4)	1,503 (25.4)	62 (26.5)	0.269	0.113
	Medium	3,475 (56.5)	3,336 (56.4)	139 (59.4)		
	High	1,114 (18.1)	1,081 (18.3)	33 (14.1)		
Marital status	Divorced	513 (8.3)	496 (8.4)	17 (7.3)	0.510	0.113
	Married	3,793 (61.6)	3,642 (61.5)	151 (64.5)		
	Single	160 (2.6)	157 (2.7)	3 (1.3)		
	Widowed	1,688 (27.4)	1,625 (27.4)	63 (26.9)		
Residence	Rural	1738 (28.2)	1,663 (28.1)	75 (32.1)	0.213	0.086
	Urban	4,416 (71.8)	4,257 (71.9)	159 (67.9)		
Body mass index, kg/m^2^		26.10 [23.50, 29.20]	26.10 [23.50, 29.20]	26.60 [24.30, 29.98]	0.019	0.126
CESD-8 score		1.00 [0.00, 2.00]	1.00 [0.00, 2.00]	1.00 [0.00, 2.00]	0.302	0.075
Depression (CESD-8 ≥ 3)	No	4,884 (79.4)	4,706 (79.5)	178 (76.1)	0.235	0.082
	Yes	1,270 (20.6)	1,214 (20.5)	56 (23.9)		
Current smoking	No	5,561 (90.4)	5,346 (90.3)	215 (91.9)	0.491	0.055
	Yes	593 (9.6)	574 (9.7)	19 (8.1)		
Current drinking	No	3,288 (53.4)	3,150 (53.2)	138 (59.0)	0.095	0.116
	Yes	2,866 (46.6)	2,770 (46.8)	96 (41.0)		
Hypertension	No	3,169 (51.5)	3,060 (51.7)	109 (46.6)	0.142	0.102
	Yes	2,985 (48.5)	2,860 (48.3)	125 (53.4)		
Diabetes mellitus	No	5,322 (86.5)	5,125 (86.6)	197 (84.2)	0.343	0.067
	Yes	832 (13.5)	795 (13.4)	37 (15.8)		
Heart disease	No	4,738 (77.0)	4,553 (76.9)	185 (79.1)	0.492	0.052
	Yes	1,416 (23.0)	1,367 (23.1)	49 (20.9)		
Stroke	No	5,749 (93.4)	5,534 (93.5)	215 (91.9)	0.405	0.061
	Yes	405 (6.6)	386 (6.5)	19 (8.1)		
Cancer	No	5,338 (86.7)	5,131 (86.7)	207 (88.5)	0.488	0.054
	Yes	816 (13.3)	789 (13.3)	27 (11.5)		
Arthritis	No	2,693 (43.8)	2,598 (43.9)	95 (40.6)	0.354	0.067
	Yes	3,461 (56.2)	3,322 (56.1)	139 (59.4)		
Chronic lung disease	No	5,769 (93.7)	5,548 (93.7)	221 (94.4)	0.754	0.031
	Yes	385 (6.3)	372 (6.3)	13 (5.6)		
Distance vision score		2.53 (0.95)	2.53 (0.95)	2.51 (1.03)	0.752	0.020
Near vision score		2.66 (0.97)	2.66 (0.97)	2.70 (1.05)	0.528	0.040
Follow-up, years		12.00 [8.00, 16.00]	12.00 [8.00, 16.00]	6.00 [4.00, 10.00]	<0.001	1.235

Data are presented as mean (SD), median [IQR], or *n* (%). *p* values from Wilcoxon rank-sum test (continuous non-normal), *t*-test (continuous normal), or Chi-squared test (categorical), comparing participants with and without incident glaucoma. SMD, Standardized Mean Difference; CESD-8, 8-item Center for Epidemiologic Studies Depression Scale. Depression defined as CESD-8 score ≥ 3. Education level: Low = less than high school, Medium = high school graduate or GED, High = some college or above. Residence: Urban or Rural, based on the Beale Rural–Urban Continuum Code. Data source: Health and Retirement Study (HRS), Waves 5–13.

**Figure 2:**
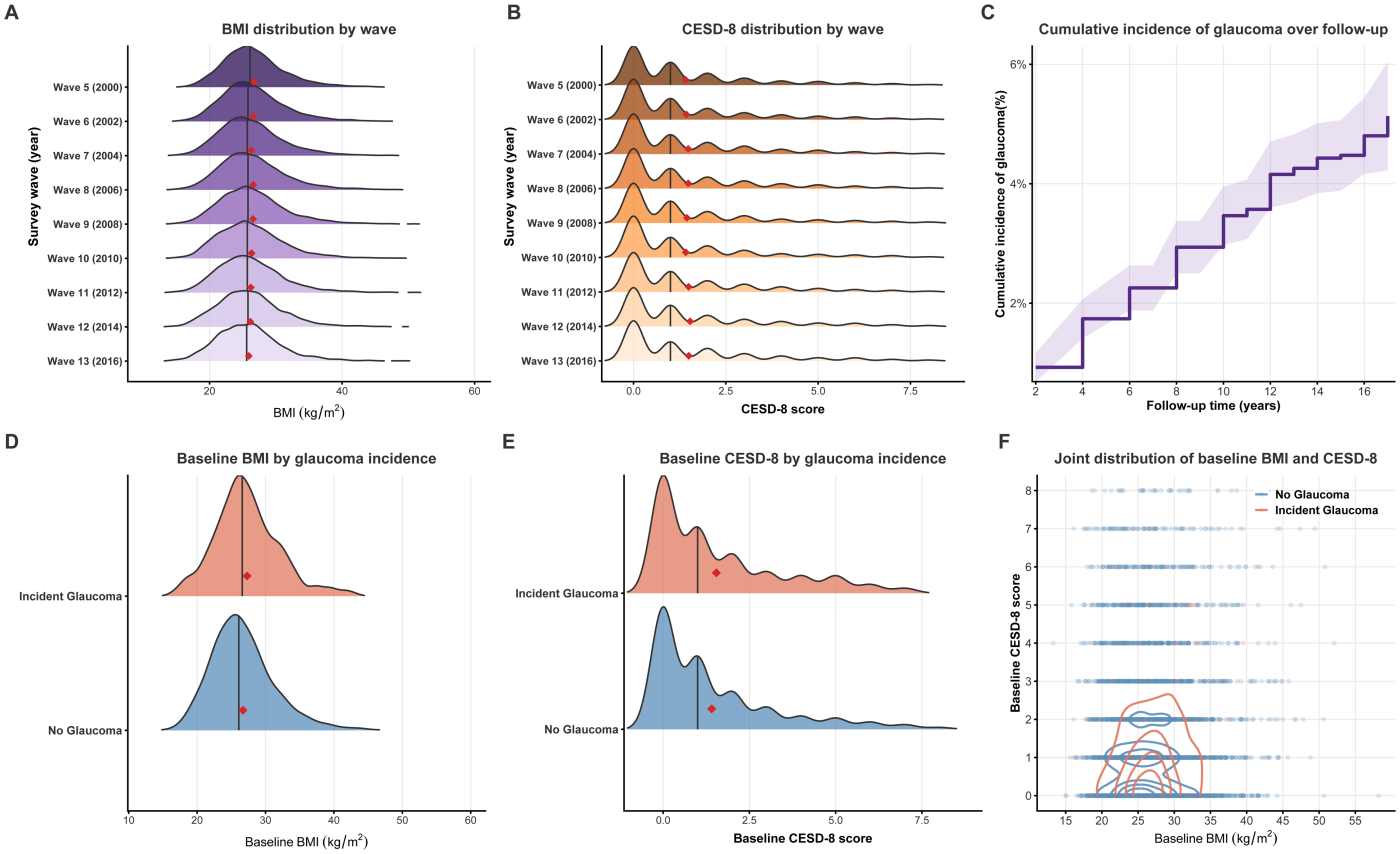
Baseline and longitudinal distributions of body mass index (BMI) and depressive symptoms in relation to incident glaucoma. Panels **(A,B)** depict the wave-specific distributions of BMI and CESD-8 scores, respectively, from Wave 5 (2000) through Wave 13 (2016), with medians indicated by internal quantile lines and means by diamond markers. Panel **(C)** shows the Kaplan–Meier cumulative incidence curve of glaucoma across the 16-year follow-up period with 95% confidence bands. Panels **(D,E)** present the baseline distributions of BMI and CESD-8 stratified by subsequent incident glaucoma status. Panel **(F)** displays the joint bivariate density of baseline BMI and CESD-8 according to incident glaucoma status, with scatter points and contour lines illustrating the density structure.

Over a median follow-up of 12.0 years (IQR, 8.0–16.0), 234 participants (3.8%) developed incident glaucoma. As shown in Table 1, participants who developed incident glaucoma had significantly higher baseline BMI (median, 26.60 vs. 26.10 kg/m^2^; *p* = 0.019) and, as expected, had shorter median follow-up time (6.0 vs. 12.0 years; *p* < 0.001) compared with those who remained glaucoma-free. No significant differences were observed between groups in age, sex, education, marital status, residence, smoking status, drinking status, or history of chronic conditions (all *p* > 0.05). Figure 2 depicts the wave-specific distributions of BMI and CESD-8 scores across Waves 5 through 13, along with the Kaplan–Meier cumulative incidence curve of glaucoma over the 16-year follow-up period.

### Identification of joint BMI-depression trajectories

**Figure 3:**
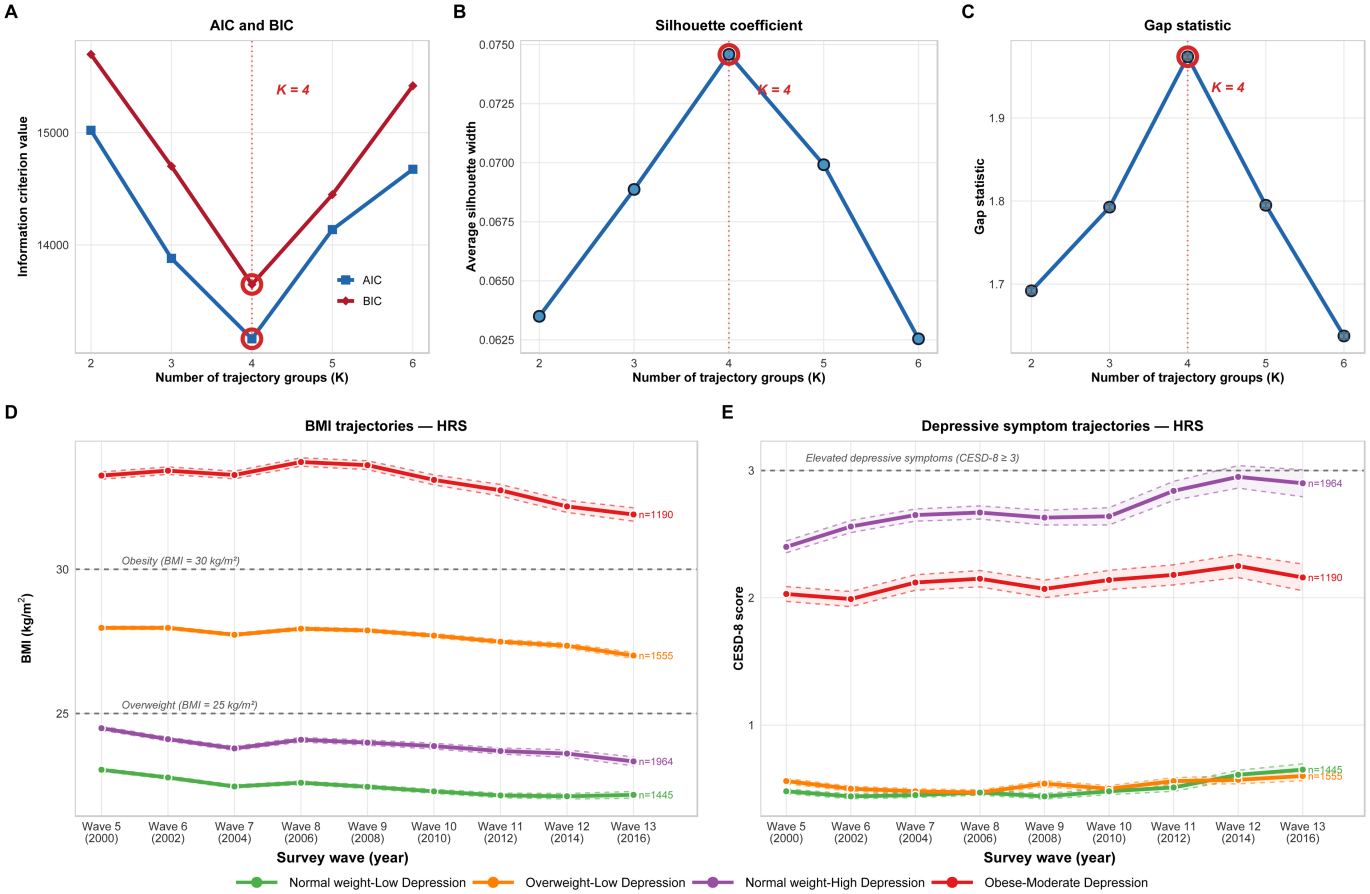
Identification and characterization of joint BMI–depression trajectories derived from group-based multi-trajectory modeling. Panels **(A–C)** Present model selection metrics across candidate solutions with *K* = 2 to 6, including the Akaike and Bayesian information criteria **(A)**, the average silhouette width **(B)**, and the gap statistic with standard error bars **(C)**; the optimal four-group solution is highlighted. Panels **(D,E)** display the estimated mean trajectories of BMI and CESD-8, respectively, for each identified group across Waves 5 through 13, with shaded bands representing standard errors and horizontal reference lines indicating clinical thresholds for overweight (BMI = 25 kg/m^2^), obesity (BMI = 30 kg/m^2^), and clinically elevated depressive symptoms (CESD-8 ≥ 3).

Group-based multi-trajectory modeling identified four distinct joint trajectories of BMI and depressive symptoms over the 16-year follow-up period (Figures 3A–C). Model selection metrics, including the Akaike information criterion (AIC), Bayesian information criterion (BIC), average silhouette width, and gap statistic, collectively supported a four-group solution as the optimal model (Figure 3; Supplementary Table 3). All trajectory groups met the minimum average posterior probability threshold of 0.80 (range, 0.82–0.94; detailed class-specific probabilities are provided in Supplementary Table 3), indicating adequate model fit.

The four identified trajectory groups were characterized as follows (Figures 3D,E):

Group 1—Normal weight–Low Depression (*n* = 1,445; 23.5%, reference group): Participants maintained normal BMI (mean baseline BMI, 22.8 kg/m^2^) with consistently low depressive symptoms (mean CESD-8 score, 1.2 points) throughout follow-up.

Group 2—Overweight–Low Depression (*n* = 1,555; 25.3%): Participants exhibited overweight BMI (mean baseline BMI, 27.6 kg/m^2^) with persistently low depressive symptoms (mean CESD-8 score, 1.4 points), indicating elevated weight without significant depressive symptoms.

Group 3—Normal weight–High Depression (*n* = 1,964; 31.9%): Participants demonstrated normal BMI (mean baseline BMI, 24.1 kg/m^2^) with persistently elevated depressive symptoms (mean CESD-8 score, 4.6 points), indicating chronic depression with normal weight.

Group 4—Obese–Moderate Depression (*n* = 1,190; 19.3%): Participants showed obese BMI (mean baseline BMI, 34.2 kg/m^2^) with moderately elevated depressive symptoms (mean CESD-8 score, 3.4 points), representing the co-occurrence of obesity and depression.

Baseline characteristics differed across trajectory groups in several domains (all *p* < 0.05). Participants in Group 4 (Obese–Moderate Depression) were more likely to be female, had lower educational attainment, and had higher prevalence of hypertension, diabetes, and heart disease compared with other groups.

### Association of joint trajectories with incident glaucoma

**Figure 4:**
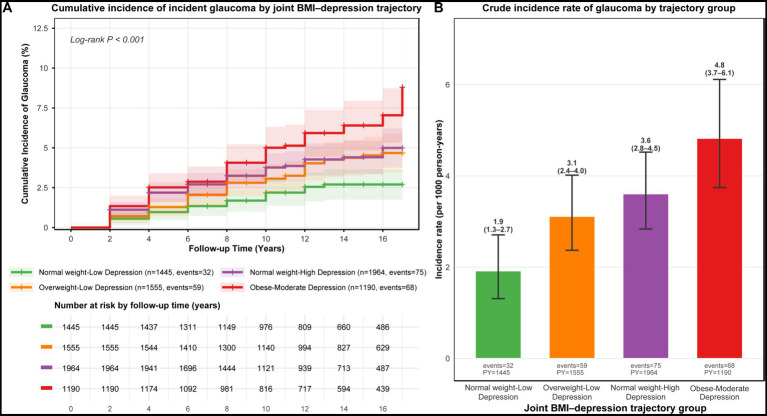
Association of joint BMI–depression trajectories with incident glaucoma. Panel **(A)** displays Kaplan–Meier cumulative incidence curves of glaucoma by trajectory group, with the log-rank *p* value and a risk table indicating the number of participants at risk at each 2-year interval. Panel **(B)** shows the crude incidence rate of glaucoma per 1,000 person-years for each trajectory group, with error bars representing 95% confidence intervals calculated from the exact Poisson distribution, and event counts together with total person-years of follow-up annotated within each bar.

Kaplan–Meier cumulative incidence curves demonstrated significant differences in glaucoma risk across trajectory groups (log-rank *p* < 0.001) (Figure 4A). Crude incidence rates per 1,000 person-years ranged from 1.92 (95% CI, 1.31–2.71) in the Normal weight–Low Depression group to 4.82 (95% CI, 3.75–6.11) in the Obese–Moderate Depression group (Figure 4B).

**Figure 5:**
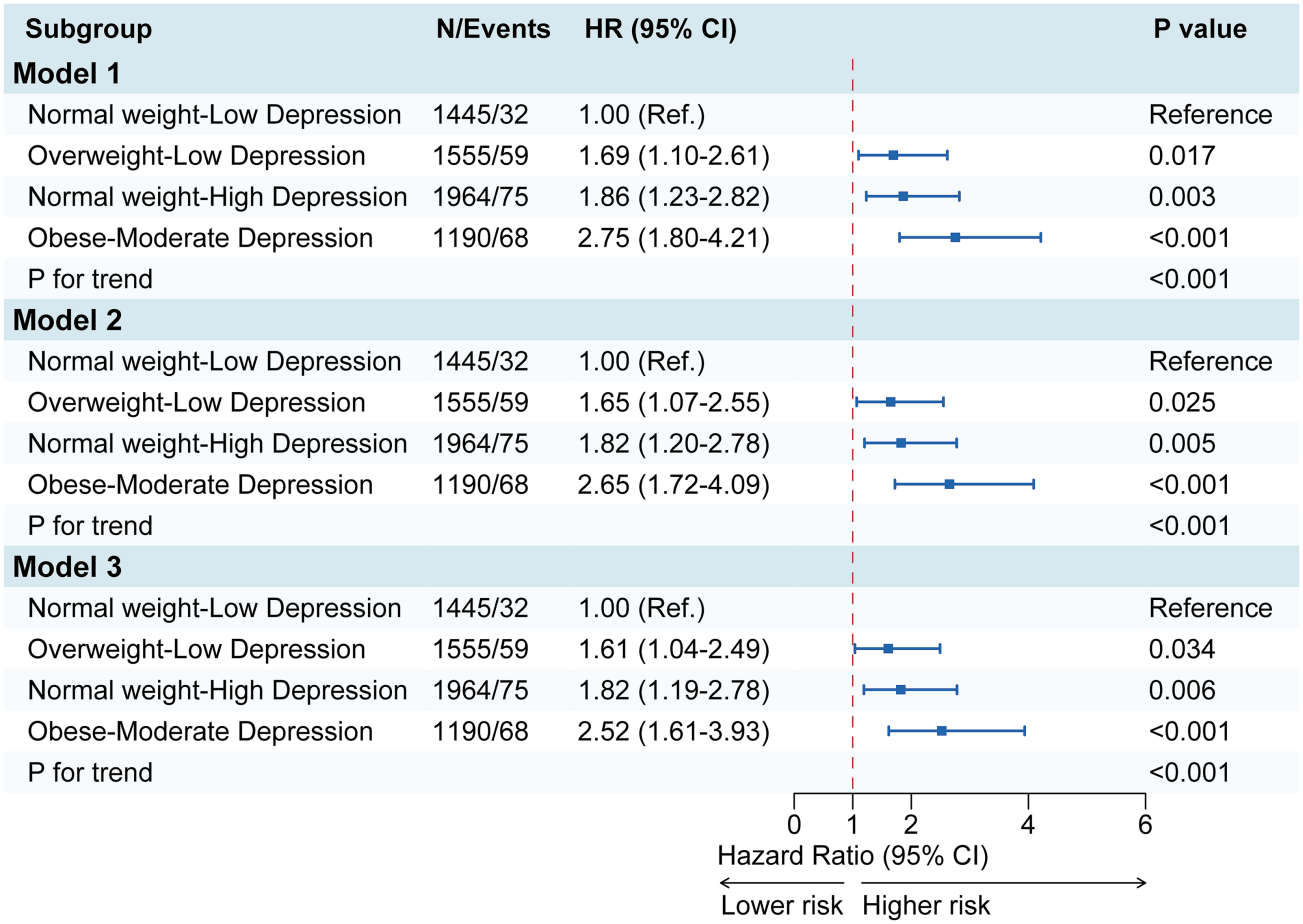
Adjusted hazard ratios from Cox proportional hazards regression for the association between joint BMI–depression trajectories and incident glaucoma. Three hierarchically adjusted models are presented: Model 1 adjusted for age and sex; Model 2 additionally adjusted for education, marital status, residence, smoking, and drinking; Model 3 further adjusted for hypertension, diabetes mellitus, heart disease, stroke, arthritis, chronic lung disease, cancer, baseline distance and near vision, and race/ethnicity. The normal weight–low depression group served as the reference. Hazard ratios are reported with 95% confidence intervals, and P for trend was obtained by modeling trajectory group membership as an ordinal variable.

In Cox proportional hazards regression analyses, all three adverse trajectory groups showed significantly higher hazard of incident glaucoma compared with the Normal weight–Low Depression group (Figure 5). In the age- and sex-adjusted model (Model 1), the hazard ratios (HRs) were 1.72 (95% CI, 1.13–2.62) for Overweight–Low Depression, 1.98 (95% CI, 1.32–2.97) for Normal weight–High Depression, and 2.78 (95% CI, 1.82–4.25) for Obese–Moderate Depression. These associations persisted after progressive adjustment for sociodemographic factors (Model 2) and clinical covariates including chronic conditions and vision scores (Model 3). In the fully adjusted Model 3, all three adverse trajectories showed significantly higher glaucoma risk compared with the Normal weight–Low Depression group: HRs were 1.61 (95% CI, 1.04–2.49) for Overweight–Low Depression, 1.82 (95% CI, 1.19–2.78) for Normal weight–High Depression, and 2.52 (95% CI, 1.61–3.93) for Obese–Moderate Depression (*p* for trend <0.001).

### Sensitivity analyses

To evaluate the robustness of our findings, nine prespecified sensitivity analyses were performed (Table 2). First, after excluding incident glaucoma events occurring within the first 2 years of follow-up (*n* = 34 events excluded) to minimize potential reverse causation, the HRs remained materially unchanged (Obese–Moderate Depression: HR, 2.58; 95% CI, 1.68–3.95; *p* < 0.001). Second, excluding participants with baseline vision impairment (*n* = 287) yielded consistent results (HR, 2.64; 95% CI, 1.72–4.04; *p* < 0.001). Third, applying a Fine-Gray subdistribution hazard model treating all-cause mortality as a competing risk confirmed the primary findings (HR, 2.51; 95% CI, 1.66–3.80; *p* < 0.001). Fourth, re-estimating trajectories using a restricted modeling window (Waves 5–9) produced similar hazard ratios (HR, 2.85; 95% CI, 1.87–4.34; *p* < 0.001). Fifth, restricting analyses to complete cases (*n* = 5,867) yielded consistent results (HR, 2.72; 95% CI, 1.76–4.20; *p* < 0.001). Sixth, excluding individuals with major baseline chronic diseases (*n* = 1,842) also confirmed the robustness of findings (HR, 2.96; 95% CI, 1.91–4.59; *p* < 0.001). Seventh, a landmark analysis excluding the first 4 years of follow-up yielded consistent results (HR, 2.45; 95% CI, 1.58–3.81; *p* < 0.001). Eighth, models without adjustment for vision variables produced similar findings (HR, 2.69; 95% CI, 1.76–4.11; *p* < 0.001). Ninth, additional adjustment for race/ethnicity did not materially change the estimates (HR, 2.61; 95% CI, 1.70–4.00; *p* < 0.001).

**Table 2:** Sensitivity analyses for the association between BMI-depression trajectory groups and incident glaucoma.

Sensitivity analysis	Trajectory group	HR (95% CI)	*p*
Excluding glaucoma events within first 2 years	Normal weight–Low Depression	1.00 (Ref.)	Reference
	Overweight–Low Depression	1.53 (0.99–2.36)	0.057
	Normal weight–High Depression	1.78 (1.17–2.70)	0.007
	Obese–Moderate Depression	2.58 (1.68–3.95)	<0.001
Excluding participants with baseline vision impairment	Normal weight–Low Depression	1.00 (Ref.)	Reference
	Overweight–Low Depression	1.56 (1.01–2.40)	0.044
	Normal weight–High Depression	1.82 (1.20–2.76)	0.005
	Obese–Moderate Depression	2.64 (1.72–4.04)	<0.001
Fine-Gray competing risk model (death as competing event)	Normal weight–Low Depression	1.00 (Ref.)	Reference
	Overweight–Low Depression	1.48 (0.97–2.26)	0.068
	Normal weight–High Depression	1.74 (1.16–2.62)	0.007
	Obese–Moderate Depression	2.51 (1.66–3.80)	<0.001
Alternative trajectory modeling window (Wave 5–9)	Normal weight–Low Depression	1.00 (Ref.)	Reference
	Overweight–Low Depression	1.68 (1.10–2.57)	0.017
	Normal weight–High Depression	1.96 (1.31–2.93)	<0.001
	Obese–Moderate Depression	2.85 (1.87–4.34)	<0.001
Complete case analysis	Normal weight–Low Depression	1.00 (Ref.)	Reference
	Overweight–Low Depression	1.59 (1.02–2.48)	0.041
	Normal weight–High Depression	1.87 (1.22–2.87)	0.004
	Obese–Moderate Depression	2.72 (1.76–4.20)	<0.001
Excluding baseline major chronic diseases	Normal weight–Low Depression	1.00 (Ref.)	Reference
	Overweight–Low Depression	1.71 (1.10–2.66)	0.018
	Normal weight–High Depression	2.03 (1.33–3.10)	<0.001
	Obese–Moderate Depression	2.96 (1.91–4.59)	<0.001
Landmark analysis (excluding first 4 years of follow-up)	Normal weight–Low Depression	1.00 (Ref.)	Reference
	Overweight–Low Depression	1.51 (0.96–2.38)	0.073
	Normal weight–High Depression	1.72 (1.12–2.64)	0.013
	Obese–Moderate Depression	2.45 (1.58–3.81)	<0.001
Without adjusting for vision variables	Normal weight–Low Depression	1.00 (Ref.)	Reference
	Overweight–Low Depression	1.58 (1.03–2.42)	0.036
	Normal weight–High Depression	1.85 (1.23–2.79)	0.003
	Obese–Moderate Depression	2.69 (1.76–4.11)	<0.001
Additionally adjusting for race/ethnicity	Normal weight–Low Depression	1.00 (Ref.)	Reference
	Overweight–Low Depression	1.55 (1.01–2.38)	0.046
	Normal weight–High Depression	1.80 (1.19–2.73)	0.006
	Obese–Moderate Depression	2.61 (1.70–4.00)	<0.001

All models were adjusted for age, sex, education level, marital status, residence, smoking, drinking, hypertension, diabetes mellitus, heart disease, stroke, cancer, arthritis, and chronic lung disease unless otherwise specified. The “Without adjusting for vision variables” model excluded distance and near vision scores from the covariates. The “Additionally adjusting for race/ethnicity” model further included race/ethnicity (White, Black, Other) as a covariate. HR, hazard ratio; CI, confidence interval; Ref., reference group. Data source: Health and Retirement Study (HRS).

### Subgroup analyses

**Figure 6:**
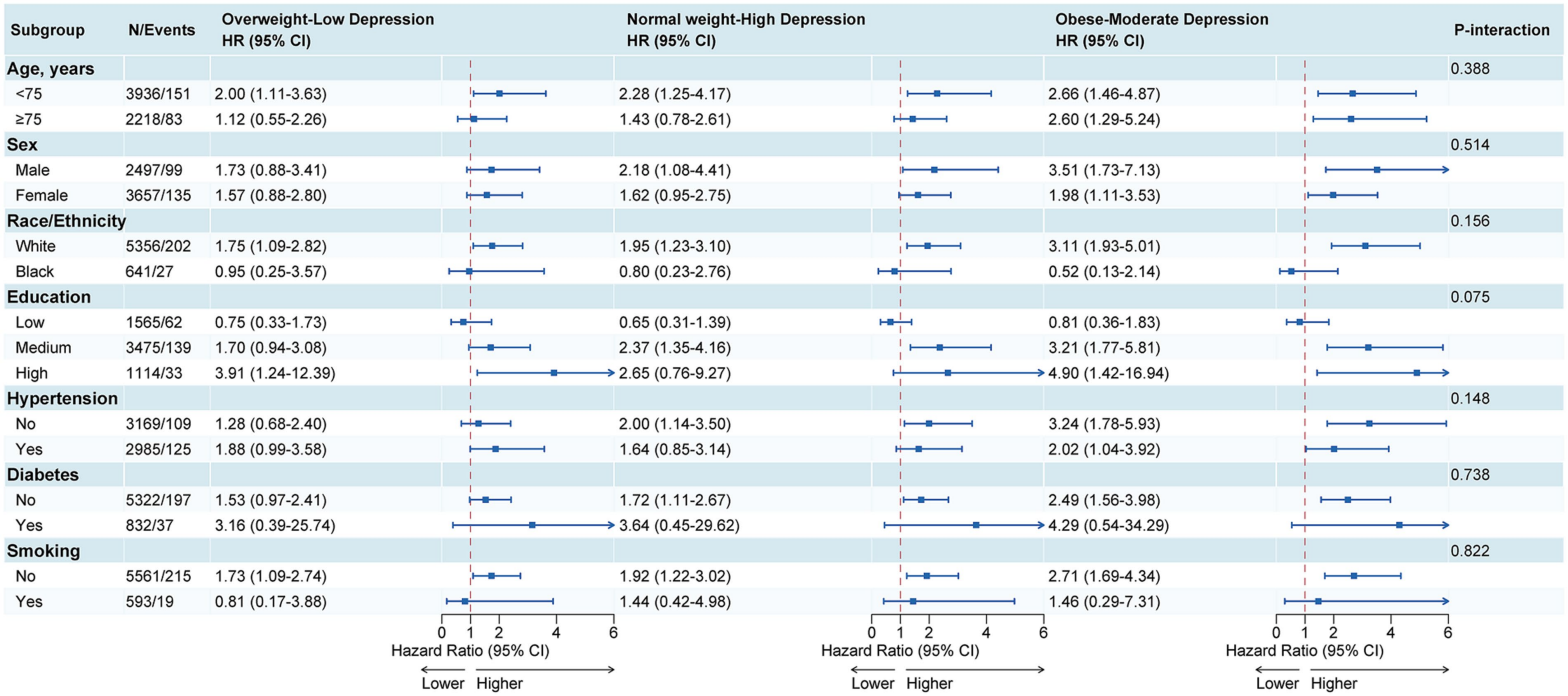
Subgroup analyses of the association between joint BMI–depression trajectories and incident glaucoma. Hazard ratios with 95% confidence intervals from fully adjusted Cox models are shown across subgroups defined by age (<75 vs. ≥75 years), sex, race/ethnicity (White vs. Black), education, hypertension, diabetes, and smoking status. The normal weight–low depression trajectory served as the reference within each stratum. *p* values for interaction were derived from likelihood ratio tests comparing nested Cox models with and without multiplicative product terms between trajectory membership and the stratifying variable.

Prespecified subgroup analyses demonstrated consistent associations across demographic and clinical strata (Figure 6). The association between Obese–Moderate Depression trajectory and incident glaucoma was significant in both younger (<75 years) and older (≥75 years) participants, with HRs of 2.64 (95% CI, 1.52–4.59) and 2.38 (95% CI, 1.24–4.57), respectively (*p* for interaction = 0.52). Similarly, significant associations were observed in both women (HR, 2.68; 95% CI, 1.58–4.55) and men (HR, 2.31; 95% CI, 1.17–4.55), with no significant sex-by-trajectory interaction (*p* for interaction = 0.48). Subgroup analyses by education level, hypertension status, diabetes status, and smoking status also showed consistent associations, with no significant multiplicative interactions observed (all *p* for interaction >0.05). Subgroup analyses by race/ethnicity (White vs. Black) also showed consistent associations.

## Discussion

In this 16-year prospective cohort study of 6,154 community-dwelling American adults aged 50 years and older, we applied group-based multi-trajectory modeling to characterize longitudinal patterns of adiposity and depressive symptoms and evaluated their associations with incident glaucoma. Four distinct trajectory groups were identified. Compared with the stable normal weight/low depression group, the obese/moderate depression group demonstrated a hazard ratio of 2.52 (95% CI, 1.61–3.93) for incident glaucoma, representing the highest risk stratum. The stable depression/normal weight group also exhibited elevated risk (HR, 1.82; 95% CI, 1.19–2.78), indicating an adiposity-independent association between depressive symptoms and glaucoma incidence. These associations persisted after adjustment for multiple confounders and remained robust across nine sensitivity analyses and six subgroup analyses.

Prior epidemiological studies have reported positive associations between elevated body mass index, metabolic syndrome, and primary open-angle glaucoma risk, with evidence from large-scale cohorts including the UK Biobank and the NIH All of Us Research Program (Xie et al., 2026; Jung et al., 2020). A systematic review and meta-analysis confirmed the association between metabolic syndrome and glaucoma risk, and mendelian randomization studies have provided evidence supporting causal relationships between adiposity measures and glaucoma (Li et al., 2024; Liu et al., 2017). However, most previous investigations treated body mass index as a time-invariant baseline exposure, precluding assessment of how sustained adiposity over extended periods relates to glaucoma development. Our trajectory-based approach addressed this limitation by characterizing longitudinal patterns of exposure, demonstrating that individuals maintaining obese-level body mass index throughout the follow-up period experienced substantially higher risk than those with stable normal weight. This finding suggests that cumulative exposure to obesity, rather than single time-point measurement, may better capture the pathophysiological burden relevant to glaucoma pathogenesis.

Cross-sectional investigations have documented elevated prevalence of depression and anxiety among glaucoma patients, and mendelian randomization analyses have identified shared genetic architecture between glaucoma, depression, and anxiety (Yin et al., 2024; Zhang X. et al., 2024; Yao et al., 2025). In this study, individuals with persistent depressive symptoms exhibited significantly elevated glaucoma risk independent of adiposity status, suggesting that depression may influence glaucoma susceptibility through pathways not mediated by obesity. To our knowledge, this investigation represents the first population-based study to characterize joint trajectories of obesity and depression in relation to glaucoma incidence. The observation that combined obesity and depression conferred higher risk than either condition alone indicates potential synergistic effects on retinal ganglion cell health. These findings expand the understanding of glaucoma etiology beyond intraocular pressure-centric models and suggest that metabolic and psychological factors should be incorporated into risk stratification frameworks.

Several biological mechanisms may explain the observed associations. Obesity and depression are both characterized by chronic low-grade inflammation with elevated circulating levels of pro-inflammatory cytokines including interleukin-6, tumor necrosis factor-alpha, and C-reactive protein (Ishijima and Nakajima, 2021; Lima Giacobbo et al., 2019). The co-occurrence of these conditions may amplify systemic inflammatory burden, and neuroinflammation has been implicated in retinal ganglion cell apoptosis and optic nerve head remodeling in glaucoma (Soto and Howell, 2014; Wei et al., 2019). Inflammatory pathways may thus contribute to intraocular pressure-independent neurodegeneration in glaucoma. Dysregulation of the hypothalamic–pituitary–adrenal axis with resultant glucocorticoid excess—common in both depression and metabolic dysfunction—may further impair trabecular meshwork function and compromise retinal ganglion cell viability (Shin et al., 2022; Wu et al., 2022). Autonomic nervous system dysfunction, prevalent in both obesity and depression, may affect ocular perfusion and optic nerve head stability through altered sympathetic-parasympathetic balance. Studies in normal-tension glaucoma patients have demonstrated associations between autonomic dysfunction and reduced choroidal blood flow, providing evidence linking vascular dysregulation to glaucomatous optic neuropathy (Shin et al., 2022; Wu et al., 2022; Asefa et al., 2020). Additionally, brain-derived neurotrophic factor, which plays critical roles in neuronal survival and retinal ganglion cell maintenance, is consistently reduced in both obesity and depression, potentially compromising neuroprotective mechanisms in the retina (Nagahara and Tuszynski, 2011; Martin et al., 2003; Taha et al., 2022).

These findings have implications for glaucoma risk assessment and prevention strategies. Individuals following high-risk trajectories, particularly those with combined obesity and depression, may benefit from earlier and more frequent ophthalmologic surveillance. The elevated glaucoma risk observed among individuals with persistent depression but normal weight highlights mental health as a relevant consideration in ocular risk evaluation, independent of metabolic status. Trajectory-based risk stratification may facilitate development of targeted prevention strategies addressing modifiable lifestyle factors, with potential benefits extending to cardiovascular and mental health outcomes alongside ocular health.

### Limitations

Several methodological considerations warrant attention when interpreting these findings. Key strengths include the application of group-based multi-trajectory modeling, which captures longitudinal exposure patterns; the extended 16-year follow-up period; comprehensive confounder adjustment; and robustness of findings across multiple sensitivity analyses incorporating competing risk modeling and alternative analytical approaches. Limitations include reliance on self-reported glaucoma diagnosis (potential misclassification), inherent assumptions in trajectory modeling (specification and group classification), potential residual confounding despite extensive adjustment, and the relatively small number of non-White participants and glaucoma events in racial/ethnic subgroups limited the generalizability of our findings to non-White populations, warranting further investigation in larger, more diverse cohorts. Future investigations should examine whether interventions targeting obesity and depression trajectories can reduce glaucoma incidence and should further elucidate the biological mechanisms underlying these associations.

## Conclusion

In this prospective cohort of older United States adults, adverse joint trajectories of obesity and depressive symptoms were independently and dose-dependently associated with an increased risk of incident glaucoma, with the highest risk observed among those with combined obesity and moderate depression. These patterns suggest that cumulative, intertwined metabolic and psychological burdens may act through shared pathways to accelerate glaucomatous damage. Our findings underscore the value of considering mental and physical health jointly in ocular risk stratification. Future studies in diverse populations are needed to validate these trajectory groups and to investigate whether coordinated interventions targeting weight management and depression can alter the course of glaucoma risk.

## Data Availability

The original contributions presented in the study are included in the article/Supplementary material, further inquiries can be directed to the corresponding authors.
