# Supplementary material for: Joint trajectories of obesity and depressive symptoms and risk of incident glaucoma: a 16-year prospective cohort study from the Health and Retirement Study
Source: Front Aging Neurosci. 2026 Aug 3;18:1910500. doi: 10.3389/fnagi.2026.1910500 (PMC13478263; doi:10.3389/fnagi.2026.1910500)
Supplement: Supplementary file 1 [file Data_Sheet_1.DOCX]

**Supplementary Table 1.** Comparison of Baseline Characteristics Between Included and Excluded Participants

| **Characteristics** | **level** | **Included (n=6,154)** | **Excluded (n=13,424)** | **P** |
| --- | --- | --- | --- | --- |
| Age, years |  | 72.00 [68.00, 77.00] | 73.00 [68.00, 79.00] | 0.062 |
| Sex | Female | 3,657 (59.4) | 7,921 (59.0) | 0.573 |
|  | Male | 2,497 (40.6) | 5,503 (41.0) |  |
| Race/Ethnicity | White | 5,356 (87.0) | 11,548 (86.0) | 0.138 |
|  | Black | 641 (10.4) | 1,477 (11.0) |  |
|  | Other | 157 (2.6) | 399 (3.0) |  |
| Education level | Low | 1,565 (25.4) | 3,573 (26.6) | 0.152 |
|  | Medium | 3,475 (56.5) | 7,489 (55.8) |  |
|  | High | 1,114 (18.1) | 2,362 (17.6) |  |
| Marital status | Divorced | 513 (8.3) | 1,155 (8.6) | 0.487 |
|  | Married | 3,793 (61.6) | 8,147 (60.7) |  |
|  | Single | 160 (2.6) | 390 (2.9) |  |
|  | Widowed | 1,688 (27.4) | 3,732 (27.8) |  |
| Residence | Rural | 1,738 (28.2) | 3,893 (29.0) | 0.265 |
|  | Urban | 4,416 (71.8) | 9,531 (71.0) |  |
| Current smoking | No | 5,561 (90.4) | 12,028 (89.6) | 0.098 |
|  | Yes | 593 (9.6) | 1,396 (10.4) |  |
| Current drinking | No | 3,288 (53.4) | 7,300 (54.4) | 0.192 |
|  | Yes | 2,866 (46.6) | 6,124 (45.6) |  |
| Hypertension | No | 3,169 (51.5) | 6,764 (50.4) | 0.143 |
|  | Yes | 2,985 (48.5) | 6,660 (49.6) |  |
| Diabetes mellitus | No | 5,322 (86.5) | 11,492 (85.6) | 0.088 |
|  | Yes | 832 (13.5) | 1,932 (14.4) |  |
| Heart disease | No | 4,738 (77.0) | 10,178 (75.8) | 0.068 |
|  | Yes | 1,416 (23.0) | 3,246 (24.2) |  |
| Stroke | No | 5,749 (93.4) | 12,425 (92.6) | 0.028 |
|  | Yes | 405 (6.6) | 999 (7.4) |  |
| Cancer | No | 5,338 (86.7) | 11,570 (86.2) | 0.316 |
|  | Yes | 816 (13.3) | 1,854 (13.8) |  |
| Arthritis | No | 2,693 (43.8) | 5,817 (43.3) | 0.555 |
|  | Yes | 3,461 (56.2) | 7,607 (56.7) |  |
| Chronic lung disease | No | 5,769 (93.7) | 12,470 (92.9) | 0.029 |
|  | Yes | 385 (6.3) | 954 (7.1) |  |
| Distance vision score |  | 2.53 (0.95) | 2.49 (0.98) | 0.312 |
| Near vision score |  | 2.66 (0.97) | 2.62 (1.00) | 0.427 |

**Supplementary Table 2.** Definitions and Coding of Study Variables

| **Variable** | **Data Source (HRS)** | **Definition / Coding** |
| --- | --- | --- |
| Incident glaucoma | Self-reported physician diagnosis | 0 = No; 1 = Yes (first reported glaucoma diagnosis during follow-up, Waves 6–13) |
| Age | Baseline (Wave 5) | Continuous, years |
| Sex | Baseline | Male; Female |
| Race/Ethnicity | Baseline | White; Black; Other |
| Education level | Baseline | Low = less than high school; Medium = high school graduate or GED; High = some college or above |
| Marital status | Baseline | Married; Divorced; Widowed; Single |
| Residence | Baseline | Urban; Rural (based on Beale Rural-Urban Continuum Code) |
| BMI | Baseline, self-reported height and weight | Continuous, kg/m²; calculated as weight(kg) / height(m)² |
| CESD-8 score | Baseline | Continuous, range 0–8; 8-item Center for Epidemiologic Studies Depression Scale |
| Depression | Derived from CESD-8 | 0 = No (CESD-8 < 3); 1 = Yes (CESD-8 ≥ 3) |
| Current smoking | Baseline | No; Yes |
| Current drinking | Baseline | No; Yes |
| Hypertension | Self-reported physician diagnosis | 0 = No; 1 = Yes |
| Diabetes mellitus | Self-reported physician diagnosis | 0 = No; 1 = Yes |
| Heart disease | Self-reported physician diagnosis | 0 = No; 1 = Yes |
| Stroke | Self-reported physician diagnosis | 0 = No; 1 = Yes |
| Cancer | Self-reported physician diagnosis | 0 = No; 1 = Yes |
| Arthritis | Self-reported physician diagnosis | 0 = No; 1 = Yes |
| Chronic lung disease | Self-reported physician diagnosis | 0 = No; 1 = Yes |
| Distance vision score | Baseline | Continuous, range 1–4; self-rated distance vision (1 = poor, 4 = excellent) |
| Near vision score | Baseline | Continuous, range 1–4; self-rated near vision (1 = poor, 4 = excellent) |
| Follow-up years | Derived | Continuous; time from baseline (Wave 5) to incident glaucoma or last available wave |

**Supplementary Table 3.** Model Fit Indices and Posterior Classification Diagnostics for Latent Class Trajectory Analysis

| **K** | **AIC** | **BIC** | **Silhouette** | **Gap Statistic** | **Optimal** | **Trajectory Group** | **N (%)** | **Mean Posterior Probability** |
| --- | --- | --- | --- | --- | --- | --- | --- | --- |
| 2 | 15,021.7 | 15,698.1 | 0.0635 | 1.6920 |  | — | — | — |
| 3 | 13,880.5 | 14,701.5 | 0.0689 | 1.7926 |  | — | — | — |
| 4 | 13,164.0 | 13,648.2 | 0.0746 | 1.9737 | Yes | Normal weight–Low Depression | 1,445 (23.5) | 0.82 |
|  |  |  |  |  |  | Normal weight–High Depression | 1,964 (31.9) | 0.88 |
|  |  |  |  |  |  | Overweight–Low Depression | 1,555 (25.3) | 0.91 |
|  |  |  |  |  |  | Obese–Moderate Depression | 1,190 (19.3) | 0.94 |
| 5 | 14,137.3 | 14,448.9 | 0.0699 | 1.7949 |  | — | — | — |
| 6 | 14,674.5 | 15,418.5 | 0.0625 | 1.6376 |  | — | — | — |

A 4-class solution was selected based on the lowest AIC and BIC values, the highest Silhouette coefficient, and the largest Gap statistic. Trajectory group assignments, proportions, and mean posterior probabilities are reported only for the optimal model (K = 4). Mean posterior probabilities for all classes exceeded the recommended threshold of 0.70, indicating acceptable classification certainty (range: 0.82–0.94; overall average: 0.89). AIC = Akaike Information Criterion; BIC = Bayesian Information Criterion.
